# Table Manners

**Published:** 1869-05

**Authors:** 


### TABLE MANNERS.

When to eat, and what, and how much, are questions which have been abundantly answered, well and ill; but it is not considered as it ought to be, that the attendants of the family table have a much larger share in promoting a healthful digestion than is generally supposed.

A good appetite is essential to a good digestion, but a snow white table cloth is a great promotive of a good appetite. No one can eat in comfort if any member of the family appears at the table in slatternly dress; with unkempt hair; showing a breadth of black under the finger nails, with a hawking and a spitting and a blowing of the nose, and their tremendous associations.

But the spotless napkin, the most splendid roast, and faultless concomitants all, what do these amount to, if sadness is written on the face of the wife; if an angry scowl gleams from the corrugated brow of a morose husband, or a dissatisfied look comes from a child's eye, and the meal is partaken of in ominous silence? Away with such unloveliness! there is no sunshine in such a household, and the members of that family, if they grow up at all, will become the refrigerators, the the bane, of every company into which they may be thrown in after life.

Rather let the family table be the place of glad reunions; as much looked forward to as the promised coming of a cherished friend; let courtesies more than courtly be ever cultivated; let smiles wreathe every face; let calm satisfaction sit on every countenance; let light hearts, and cheery words, and obliging acts, and watchful attentions be the order of the day; these are the promoters of a healthy digestion; and these are they which largely help to make happy homes, and good hearts and generous natures.

